# Remarks on Mustard Poultices, Applied Extensively to the Surface

**Published:** 1845-01

**Authors:** William O. Baldwin

**Affiliations:** Montgomery, Alabama


### WESTERN JOURNAL

OF

### MEDICINE AND SURGERY.

JANUARY, 1845.

Art. I.—*Remarks on Mustard Poultices, applied extensively to the surface.* By William O. Baldwin, M.D., of Montgomery, Alabama.

The application of poultices as a remedial agent in many forms of local inflammations, spasmodic pains, &c., has long been practised and highly appreciated, as not the least efficacious among the many sanative agents available in such cases. By some they have been and are still used with reference solely to the specific virtues of the substances of which they are composed, whilst others esteem them all for their one common virtue—attributing to them no other curative effect than that which arises from their capability of retaining warmth and moisture about the parts to which they are applied. Both of these views, as to their *modus operandi*, are probably correct; for the results which follow the endermic use of medicine undoubtedly establishes the truth of the former; whilst all can attest the good results which are frequently obtained from the application of *simple* poultices to inflamed and painful parts, which of course can be attributed to nothing more than the relaxation afforded by the warmth and moisture which they contain. It is for this property of the poultice, added to the increased revulsive effect of the mustard, when combined, that I propose to extend their use to acute diseases, involving the whole animal economy.

From the marked success which has attended the application of mustard poultices to the entire, or greater portion of the surface of the body and extremities, in the treatment of some diseases of an idiopathic character, I am disposed to regard them as a remedy of more value than their hitherto partial use would seem to indicate; for although highly esteemed and extensively employed in certain *local* affections, so far as I am aware, their application has been restricted to such diseases, or when used in those involving a greater extent of tissue they have been applied only to combat some local symptom.

The experience which I have had with the mustard leads me to look upon it as a remedy peculiarly applicable to diseases of a congestive type, and more especially to *congestive fever.* I have found it a most prompt and available remedy in one or two instances in which I have used it in *convulsions* occurring in children; and I have also applied it advantageously in some cases of *visceral inflammation.* In a case of *trismus nascentium* in which I used it, there was a considerable abatement of the disease under its influence; but as the violent symptoms afterwards returned and the case terminated fatally, it cannot be considered as furnishing any testimony in support of its efficacy in that class of diseases. I believe, however, that if my directions had been carried out during the twelve hours of remission and comparative ease which the little sufferer seemed to enjoy immediately after the application of the poultice, which would have brought it fully under the influence of opium, it might have succeeded. That, however, is a bare supposition, but I was so well pleased with the effect which followed its application, that, though the case proved fatal, taking into consideration the very unsatisfactory results which have generally followed the treatment of that disease, I shall test its efficacy further should a suitable occasion offer.

From its powerful agency in producing diaphoresis, that end so often coveted in the treatment of *synochal fevers*, I am induced to believe the mush poultice, with or without the mustard, applied in this way, might form a valuable adjuvant in the treatment of that class of diseases.

These remarks are not intended to depreciate the value of free internal medication. Indeed, in most of the cases in which I have used the poultice it has been in conjunction, or at least, not to the exclusion of any other remedy which might have been thought available—so that one who may be disposed to cavil, might with some plausibility, dispute its agency in producing the beneficial results which I have ascribed to it. Nevertheless, in a great majority of the cases in which I have resorted to it, in the diseases alluded to, its effect has been so apparent that notwithstanding the administration of other remedies, at the same time, no one could mistake its promptly favorable influence. And it frequently happens that our only means of making an impression upon the disease is through the agency of external applications. As, in *cerebral congestions* attended with coma and insensibility, it is always difficult, and sometimes even impossible, to administer medicines internally—in such cases the mustard poultice is entitled to the very first and highest consideration, as the remedy most likely to produce the desired objects.

Though this is a remedy of most singular simplicity, yet a few remarks as to the mode which I have adopted, both in its preparation and application, may not be considered inappropriate.

Supposing the patient to be an adult of the ordinary physical proportions—boil about a bushel of meal to a tolerably consistent mush, and spread upon a sheet which has been placed on a wide mattrass, the mattrass lying on a low bedstead. The mustard, which should be prepared in the meantime, by mixing three bottles with meal, in the proportion of one part of the former to five or six of the latter, and made into a thin batter by the addition of hot water, is then poured over the mush, and the whole covered by a thin sheet. The patient is then placed about the middle of the poultice, the sides of which are folded over him—completely enveloping every part except the head. The mustard applied in this way is rendered much more active, and will redden the skin when diluted in this way to one-sixth of its strength, almost as soon as cataplasms of unmixed mustard, and much quicker when applied to cool or cold extremities—consequently, if applied without being blended with some farinacious substance it would be very likely to produce vesication.

When enveloped in this way, I have never allowed any patient to remain longer than from a quarter to half an hour, but have been governed as to the length of time which they were allowed to remain altogether by the impression made upon the disease, and see no reason why they should not be kept on a much longer time, if necessary; for, when combined with the mustard in this way, it is much less apt to produce a feeling of faintness and debility than the warm bath. It will sometimes be found inconvenient to use the poultice in this way, for want of proper and necessary appliances; in the cases of young men, for instance, living in counting-houses and other places where they have but few conveniences. In such instances a less troublesome mode, and one which I have practised in a few instances, is to envelope each limb separately and cover completely the chest and abdomen, from the neck to the pubis, with the poultice prepared in the same way, and spread on large towels. The former mode is much the most powerful, and when practicable, is to be preferred; but I have found the latter to answer a very good purpose in some instances, and from the fact that it can be borne for a much longer time, and with less annoyance to the patient, I think, perhaps, it might be equally efficacious in cases of a less urgent description.

In illustration of these views, I will append a few cases which occurred in my practice, and in which I used the poultice, prepared in the manner described above.

In the first case which follows, the mustard was left out of the poultice, in consequence of a sufficient quantity not being at hand. It will also be seen in this case, that the application of sinapisms and extensive blistering had been practised previous to the use of the poultice, without producing any favorable impression on the disease.

Case I.—October 5th, 1841, I was called in consultation with Wm. Morris, to Mrs. E——, æt. about 24 years, a resident of this city, then on a visit to an adjoining county. Has coldness of the extremities; small, frequent and irregular pulse; excessive nausea, and occasional vomiting; great restlessness and thirst; complains of a sense of oppressive weight and fulness about the præcordial region; tongue white, furred and enlarged. This was the fifth day of her disease, and I was informed by Dr. M. that her symptoms were but little altered since her attack—her extremities had only been warmed by the application of artificial heat. The treatment which had been pursued consisted of mercurial and other laxatives, and stimulants internally, sinapisms to the extremities and epigastrium, &c. The bowels had been pretty freely moved—evacuations thin, and of an indigo-blue color.

The treatment agreed upon after my arrival, was sulph. quinine grs. vi., to be repeated at intervals of four hours, and hydrarg. cum creta grs.vi., pulv. opii gr.ss.; M. to be repeated every sixth hour; warm applications to the extremities.

6th. Had several motions, consisting of the same thin, serous and bluish matter, interspersed with flakes of mucus of the same color; condition in other respects the same. Same prescription continued and warm bath ordered.

7th. Condition unaltered. Applied a blister to the epigastrium and nape of the neck; ordered hydrarg. subm. grs.iv., creta prep. grs.iii., to be repeated at intervals of four hours; warm applications and occasionally sinapisms to the extremities. Discontinued quinine and other previous prescriptions.

8th. No improvement; secretions from bowels same; applied blisters to the extremities. Ordered tr. valerian ammon. ʒij. every third hour. Calomel, &c., continued.

9th. Evening.—Blisters vesicated; pulse rather more frequent, but still irregular and weak; has vomited most of the medicine taken in the last 24 hours, and her symptoms generally present rather an aggravated aspect. Fearing the natural tendency, which characterizes some of our diseases, to a crisis on the 9th day, and seeing no indication to a favorable one in this instance, we agreed to envelope the patient in a mush poultice, prepared in the mode described above, leaving out the mustard; which was accordingly soon done. The patient thus covered to the extent of an inch, or an inch and a half in mush, was allowed to remain about 18 minutes, at the end of which time, as the pulse had diminished in frequency and had become more regular, the skin on the forehead and face being covered with a warm perspiration, she was removed to bed. One hour after, her extremities were still warm, and the surface generally covered with a warm moisture, and her pulse manifestly improved in volume and regularity. Gave subm. hydrarg. grs.x. She is to take no other medicine through the night, and be allowed to sleep as much as possible.

10th. Extremities remain warm and skin soft; rested well through the night; pulse regular, and but little above a healthy standard in point of frequency, and also improved in volume; that feeling of fulness and oppression about the præcordia, which has been so distressing throughout the disease, entirely disappeared; after an enema, administered this morning, had two dark, consistent evacuations—in short, there is an abatement of all the violent symptoms, and the patient expresses herself as being much relieved. No medicine given this morning, but directed her to have chicken broth occasionally through the day.

11th. Continues to improve; no directions except as to diet.

13th. Is able to sit up in bed. After this, her convalescence was rapid.

Case II.—Extracts from notes on A—— C——, a female child, æt. about 20 months, who had been laboring for seven days under a severe attack of *pneumonia*, with *gastro-enteritic complication.*

February 27th, 1844. She continued to get more restless and uneasy until half past 2 o'clock, P.M., when she presented many apparent evidences of the moribund condition. Pulse 164, small and weak; respiration 44 in the minute, and imperfect; (too restless to admit the use of the stethoscope); eyes thrown back and fixed in their socket, with the eye-lids firmly separated; a disposition to throw the head back upon the spine, (as in opisthotonos), with some rigidity of the cervical and dorsal muscles; throwing the arms about, tossing the head, and writhing the body; clawing at the tongue, and at all the clothes that were brought in its way; extremities cold, the lower ones to a few inches above the knee. In this situation the patient was allowed to remain for sometime without a prescription, (more than the sinapisms which had been applied to the extremities) under the belief that if she was not in *articulo mortis* she was rapidly approaching it, and that all efforts to arrest the immediately fatal termination of her disease would be fruitless. Dr. Sims of this place, was attending the case with me, and we ordered, at length, a hot pediluvium, made strong with mustard, to be applied until a *mustard poultice* could be prepared, which was accordingly soon done in the usual way, and the entire surface completely enveloped. Up to this time, however, there was no improvement, and the weakness and frequency of the pulse, added to her extreme restlessness, precluded the possibility of counting it with anything like accuracy. She remained in the poultice eleven minutes, was taken out and washed in a tub of warm water, and placed in bed, where she remained quiet and apparently easy; her pulse increased in volume, and all those alarming symptoms which seemed to threaten immediate dissolution, considerably abated. Examined her two hours after—pulse 138, and manifestly increased in volume; eyes shut, appears to be sleeping quietly; surface generally warm and soft; reaction appears to be permanently established.

28th. Continued to rest well through the night. Pulse 132.

She remained in this condition for a day or two, which seemed to be so favorable, contrasted with her situation on the 37th, that we were inspired with strong hopes of her recovery. But on the evening of the next day, she passed two round worms; meteorism and tenderness of the abdomen, with retention of the urine supervened, and she died at night on the 3d of March, of the complication, her primary disease having been much abated.

Though the case in the end proved fatal, yet the agency of the poultice in allaying that truly urgent train of symptoms which supervened on the 27th was so manifest, that I am disposed to offer it as an additional proof of the power and utility of the remedy employed in this way.

Case III.—*Scarlatina.* March 19, 1844. Harrietta, a negro girl, æt. about 12 years, attended by Dr. Boling and myself. Saw her a few hours after the first symptoms of her indisposition. Was attacked with chilliness, succeeded by vomiting and febrile excitement. Pulse 147; stomach irritatable; tonsils red and tumefied; skin arid and hot; bowels constipated. Ordered subm. hydrarg. grs.x. and a diaphoretic mixture of spts. æth. nit., &c. &c.

20th. Morning. Pulse 150; had a very restless night; vomiting continues; complains of severe pain in the head. Medicine produced several motions. Diaphoretic mixture continued.

Evening. Pulse 170; tongue dry; great thirst; less irritabilty about the stomach; in other respects the same. Ordered warm bath, and decoction of serpentaria at intervals during the night.

21st. Pulse 166; has been delirious since the early part of the night; had two motions; condition same in other respects. Ordered sulph. quin. grs.vi. at 8 o'clock, to be repeated at 12 o'clock. Decoction serpentaria continued.

Evening. Condition same. Ordered sulph. quin. grs.iv., pulv. opii gr.$\frac{1}{4}$th. M.—to be repeated at intervals of four hours.

22d. Same condition; pulse 166; passed a restless night, starting, incoherent talking, &c. Quinine continued. At 11 o'clock, A.M., she was enveloped in a *mustard poultice*, allowed to remain fifteen minutes, and after being placed in a warm bath and washed, was removed to bed. Applied a blister of flies to the scalp; gave subm. hydrarg. grs.v., and continued the quinine. While she was in the poultice her forehead became moist, and on counting her pulse immediately after she was removed to bed, it was found to be reduced to 154.

Evening. Pulse reduced to 134; skin soft; more rational and calm. Quinine continued.

23d. Pulse 120; she is perfectly sensible; rested tolerably well through the night; countenance cheerful, and expresses herself as feeling much better. Quinine continued, and ordered ol. ricini ℥vi., which produced several consistent, bilious evacuations. She continued to improve slowly; desquamation commenced on the 30th and was general.

In this case there were many other remedies used, but the commencement of convalescence was marked by the salutary impression of the poultice, which was observed during its application.

Case IV.—October 6th, 1843. J—— W——, a bank officer, æt. about 27 years, was attacked four days ago with a chill, succeeded by nausea and vomiting; dry tongue; excessive thirst, great oppression about the epigastrium; pain in the head and back, and frequent pulse; skin moderately warm and dry. The treatment during this time consisted of mild mercurial and other laxatives, which produced moderate alvine evacuations, one bloodletting, sinapisms to the epigastrium, and quinine when it was thought advisable. This morning, pulse 126, and small; excessive vomiting; surface generally cool; extremities very cold, the lower ones to within a few inches of the groin; copious diaphoresis about the head and face; countenance pale and somewhat sunken, and speaks but little above a whisper. Everything given by the mouth is thrown up almost immediately. Enveloped the extremities, and covered the thorax and abdomen with mustard poultices. About an hour after their application the system appeared to rally considerably, and as the stomach had become quiet, he was ordered to take sulph. quin. grs.iv., sulph. morph. gr.¼th, made into a pill, with the ext. taraxaci q. s. The poultices were allowed to remain on about four hours, when, at his earnest request, they were removed. His pulse by this time had fallen to 92; surface generally warm; vomiting had ceased; expresses himself much relieved; says he felt a great oppression about the praecordia which is now entirely gone. Quinine was continued in six grain doses, and occasionally combined with gr. ¼th morphine, for the succeeding 24 hours, which produced extreme restlessness. This, however, subsided a few hours after the discontinuance of the remedy—from which time he continued to convalesce without taking any more medicine.

In this case, the excessive irritability of the stomach precluded the use of internal remedies, until the most urgent symptoms had greatly subsided.

Case V.—Wade, a lad, æt. about 7 years, has been sick for several days, and in the absence of Dr. Henry, the attending physician, I was called, Oct. 7th, 1843, at night. Found him with pulse 146, small and weak; deep coma and insensibility; surface generally cool; extremities very cold; clammy perspiration about the head and face. The friends had applied sinapisms to the extremities before my arrival, which, however, had failed to redden the skin. Ordered the extremities to be enveloped and the abdomen to be covered with mustard poultices. Nothing was given the patient by the mouth—he was left with the poultices on for about an hour when he spoke. A little while after, he complained of the burning caused by them, and cried to have them removed. They were, however, allowed to remain on about two hours, when they were taken off; his pulse had by that time declined to 124; the patient was rational, and the surface of his body generally warm.

In the meantime, Dr. H. arrived, and we made the following prescription for the night:—sulph. quin., hydrarg. c. creta. ää grs. xxiv. M. in chart. xii., div., one to be given every second hour. They did not extend the poultices as high up on the extremities as I ordered, or as I generally do.

I did not see the case again, but was informed that he continued comparatively comfortable through the night, and convalesced slowly, the greatest subsequent annoyance being sores on his legs, which were occasioned by the poultice having been made too strong with mustard.

In a case of *congestive* fever, treated successfully, but which is too tedious to detail at length, I kept the extremities enveloped, and the chest and abdomen covered with the mustard poultice, for forty-eight hours—reapplying them every four or five hours.

In the treatment especially of those diseases which depend upon a want of balance in the circulation, and when the structural lesion is not very extensive, the mustard poultice used as I have recommended it, if not in itself a curative remedy, will, at least, frequently be found a powerful adjuvant.

October 23, 1844.

